# Warfarin Dosage Response Related Pharmacogenetics in Chinese Population

**DOI:** 10.1371/journal.pone.0116463

**Published:** 2015-01-16

**Authors:** Siyue Li, Yuangao Zou, Xia Wang, Xunbei Huang, Yong Sun, Yuqing Wang, Li Dong, Hong Jiang

**Affiliations:** 1 Department of Laboratory Medicine, West China Hospital, Sichuan University, Chengdu, P. R. China; 2 Department of Cardiac Surgery, West China Hospital, Sichuan University, Chengdu, P. R. China; National Cancer Center, JAPAN

## Abstract

**Objectives:**

As the most frequently prescribed anticoagulant, warfarin has large inter-individual variability in dosage. Genetic polymorphisms could largely explain the differences in dosage requirement. rs9923231 (VKORC1), rs7294 (VKORC1), rs1057910 (CYP2C9), rs2108622 (CYP4F2), and rs699664 (GGCX) involved in the warfarin action mechanism and the circulatory vitamin K were selected to investigate their polymorphism characteristics and their effects on the pharmacodynamics and pharmacokinetics of warfarin in Chinese population.

**Methods:**

220 patients with cardiac valve replacement were recruited. International normalized ratio and plasma warfarin concentrations were determined. The five genetic polymorphisms were genotyping by pyro-sequencing. The relationships of maintenance dose, plasma warfarin concentration and INR were assessed among groups categorized by genotypes.

**Results:**

rs9923231 and rs7294 in VKORC1 had the analogous genotype frequencies (D’: 0.969). 158 of 220 recruited individuals had the target INR (1.5–2.5). Patients with AA of rs9923231 and CC of rs7294 required a significantly lower maintenance dose and plasma concentration than those with AG and TC, respectively. The mean weekly maintenance dose was also significantly lower in CYP2C9 rs1057910 mutated heterozygote than in patients with the wild homozygote. Eliminating the influence from environment factors (age, body weight and gender), rs9923231 and rs1057910 could explain about 32.0% of the variability in warfarin maintenance dose; rs7294 could explain 26.7% of the variability in plasma concentration. For patients with allele G of rs9923231 and allele T of rs7294, higher plasma concentration was needed to achieve the similar goal INR.

**Conclusions:**

A better understanding of the genetic variants in individuals can be the foundation of warfarin dosing algorithm and facilitate the reasonable and effective use of warfarin in Chinese.

## Introduction

Seventy-two years since its discovery, warfarin remains the mainstay of oral anticoagulant therapy [1, 2]. It is widely prescribed for the prevention and treatment of patients with pulmonary embolism, deep venous thromboembolism, atrial fibrillation and prosthetic heart valves [3]. For the narrow therapeutic index and inter-individual variation of warfarin dosing, excessive or inadequate anticoagulation may result in serious complications associated with bleeding or thromboembolism. The capricious nature of warfarin dose response is associated with demographic, environmental, clinical, and especially genetic factors [4, 5].

In 2007, the Food and Drug Administration (FDA) had released an announcement to emphasize the importance of genetic testing for initial estimate of warfarin dose [6]. Up to now, a series of genetic variations related with the pharmacodynamics and pharmacokinetics of warfarin have been reported. To obtain an adequate anticoagulant effect, subjects bearing polymorphisms in these genes require lower or higher warfarin doses than subjects bearing the wild-type genes [7]. Furthermore, different ethnic groups are also observed to have variations in polymorphism frequencies, and Chinese patients are known to be more sensitive to warfarin and require lower doses than Caucasians [3]. Genetic polymorphisms in vitamin K epoxide reductase complex 1 (VKORC1) and cytochrome P450 2C9 (CYP2C9) which had attracted broad attention could account for 30% to 40% of the dose variation [8–10]. For Chinese, haplotypes H1 and H2 of VKORC1 which related to low warfarin requirement were more prevalent than that in Caucasians and Africans. On the contrary, haplotypes, H7, H8 and H9 associated with the higher dose requirement were high frequency in Caucasians and Africans, but lower in Chinese [11]. In addition to SNPs in VKORC1 and CYP2C9, CYP4F2 rs2108622 polymorphism was found significant variability in different ethnic groups. The T allele had higher frequency in Indian and Caucasian than in Chinese and African-American populations [12]. However, few data about the relationship between CYP4F2 and warfarin dose in Chinese was revealed. Liang, et al. found that patients with CT or TT allele needed higher dose compared to CC carriers [11]. Gene studies had also detected other candidate SNPs which may associate with warfarin dose in gamma-glutamyl carboxylase (GGCX) [13, 14], epoxide hydrolase 1(EPHX1) [15, 16], calumenin (CALU), and CYP2C19 [15]. But the consequence remains controversy. In this research, five single nucleotide polymorphisms of key genes involved in the warfarin action mechanism and the circulatory vitamin K (rs9923231 in *VKORC1*, rs7294 in *VKORC1*, rs1057910 in *CYP2C9*, rs2108622 in *CYP4F2*, and rs699664 in *GGCX*) were selected to investigate their polymorphism characteristics and their effects on the pharmacodynamics and pharmacokinetics of warfarin in Chinese population.

## Materials and Methods

### Study subjects

Patients with cardiac valve replacement met the following criteria can be included in this study: 1) the Han population had been in Sichuan Basin for generations; 2) undergoing the continuous warfarin therapy for 3 month at least; 3) all of them had kept a balanced diet without smoking and drinking; 4) did not take any other medications which may interfere with the pharmacokinetics or pharmacodynamics of warfarin; 5) no laboratory evidence revealed they had hepatic or renal impairments; 6) during the warfarin therapy, no evidence and history of hemorrhage or thrombosis complications. A total of 220 patients were enrolled in this study after having obtained written informed consent. Demographics information including gender, age, body weight, and maintenance doses was collected from questionnaires. Plasma and blood samples were obtained at 12 hours after the administration of the last dose of warfarin. This study was approved by the Ethics Committee of West China Hospital, Sichuan University.

### Determination PT and INR

Blood specimens from 220 enrolled patients were collected in evacuated tubes containing 3.2% sodium citrate. The blood to anticoagulant ratio in each tube was 9:1, and the plasma was separated by a centrifugation of 4000rpm for 10 minutes. Then the PT was performed on a Sysmex CA7000 analyser (Sysmex Corp, Japan) using a Thromborel S reagent kit (Siemens Healthcare Diagnostic, Germany) and INR was calculated subsequently.

### Determination of warfarin plasma concentrations by UPLC

The plasma concentration of total warfarin was measured by Ultra performance liquid chromatography (UPLC) equipped by Waters I-class-UPLC system (Waters, UK). The column used was a 1.7μm BEH C18 (2.1×50 mm).The mobile phases consisted of 60% 0.02mol/L NH_4_Ac (PH = 3.0) and 40% acetonitrile with 0.3ml/min flow rate. The column temperature was maintained at 40°C and the detection wavelength was 308nm. 0.1ml internal standard, 0.5ml 1mol/L HCL and 3ml methyl tert-butyl ether (MTBE) were added to 0.5ml plasma sample. The mixture was thoroughly mixed for 5 minutes and then centrifuged for 3 minutes (2500r/min). The supernatant was separated and evaporated to dryness at 50°C. The residue was reconstituted with 100μl Mobile Phase and 4μl of the solution was then injected into the UPLC system. According to the peak height ratio of warfarin and internal standard, concentration quantitative analysis was performed in the standard curve.

### Genotyping by Pyrosequencing

DNA was extracted from whole blood using QIAamp DNA Blood Mini Kit (QIAGEN, UK) and then used as the template for PCR amplication. The primers for PCR and the sequencing were all designed by PyroMark Assay Design 2.0 Software. PCR was carried out on Veriti 96-Well Fast Thermal Cycler (Applied Biosystems, USA) with the following protocol: 50°C for 2 minutes; 94°C for 2 minutes; 42 cycles of 94°C for 5 seconds, 55°C or 10 seconds, 60°C for 10 seconds; final 40°C for 1 minute. The product for each sample was immobilized by master mix which contained Streptavidin Sepharose High Performance beads (GE, Healthcare, 17-5113-01) and PyroMark Binding Buffer (Qiagen, Germany. Lot No: 139295731). The immobilized product was purified by 75% ethanol, PyroMark Denaturation Solution (Qiagen, Germany. Lot No.145020111) and PyroMark Wash Buffer (Qiagen, Germany. Lot No: 145021635) on the PyroMark Q96 Vacuum Workstation (Qiagen, Germany), sequentially, and finally released in the PyroMark Q96 plate containing PyroMark Annealing Buffer (Qiagen, Germany. Lot No: 139300187) and sequencing primer. The plate with the samples was heated at 80°C for 2 minutes using a pre-warmed block, and then cooled to room temperature for at least 5 minutes. The plate was placed in the PyroMark Q96 ID Instrument (Qiagen, Germany) and pyrosequencing for 5 SNPs were performed. All SNPs were checked to ensure that they were in Hardy–Weinberg equilibrium.

### Statistical analysis

Continuous variables were expressed as mean±SD. Hardy-Weinberg equilibrium for 5 target SNPs were analyzed by online SHEsis software (http://analysis.bio-x.cn/SHEsisMain.htm). Data were analyzed using T test for normal continuous variables and Mann-Whitney U test for non-normalized continuous variables. Categorical variables were analyzed using χ2-test and fisher exact test. The effects of SNPs on warfarin maintenance dose and plasma concentration were analyzed by stepwise multiple linear regression. The maintenance dose and plasma concentration differences between the groups categorized by polymorphism genotypes were analyzed by T test and One-Way ANOVA test. Linear regression analysis was used to model the relationships of maintenance dose with plasma warfarin concentration, plasma concentration and INR. A *p*-value of less than 0.050 was considered statistically significant. All statistical analyses were performed by SPSS 17.0 software.

## Results

This study recruited two hundred and twenty patients. The demographic and clinical features including age, gender ratio, body weight, plasma warfarin concentrations, INR and maintenance dose of per week were summarized in Table 1.

**Table 1:** Characteristics of patients with cardiac valve replacement.

**Demographics**	
**Age (year) (n = 220)**	47.65±11.20
**Gender ratio (male: female) (n = 220)**	58:162
**Body weight (kg) (n = 220)**	58.47±8.46
**Plasma warfarin concentrations (ng/ml) (n = 130)**	1159.80±407.83
**INR (n = 220)**	1.83±0.49
**Maintenance dose (mg/week) (n = 220)**	20.26±7.39

Values represented as mean±SD. INR: international normalized ratio.

rs9923231 (*VKORC1*), rs7294 (*VKORC1*), rs1057910 (*CYP2C9*), rs2108622 (*CYP4F2*), and rs699664 (*GGCX*) were genotyped by pyrosequencing. The allelic and genotypic frequencies of the tested SNPs obtained in 220 subjects were shown in Table 2. The genotype distributions of those polymorphism loci were all in Hardy-Weinberg equilibrium, and were compared with those data reported in other references about Chinese population. Table 3 showed the basic information about the cited references. rs9923231 and rs7294 in *VKORC1* had the analogous genotype frequencies. The SHEsis software indicated strong evidence of linkage disequilibrium between them (D’: 0.969).

**Table 2:** Genotype and allelic frequencies of rs9923231, rs7294, rs1057910, rs2108622, rs699664 polymorphisms obtained in our population and in other references of Chinese.

	**Genotype frequencies**			**Allelic frequencies**			
**Gene**	**Our Data**		**Reference Data**		**Our Data**	**Reference Data**	
	**N**	**%**	**%**		**%**	**%**	
**VKORC1 rs9923231**							
**AA**	185	84.00	83.7–86.16	**A**	91.80	91.57–92.61	^[36][37]^
**AG**	34	15.50	12.90–15.70	**G**	8.20	7.39–8.43	
**GG**	1	0.50	0.60–0.94				
**VKORC1 rs7294**							
**TT**	1	0.50	1.00–1.45	**T**	8.00	5.31–7.30	^[20][21]^
**TC**	33	15.00	7.73–12.70	**C**	92.00	92.70–94.69	
**CC**	186	84.50	86.30–90.82				
**CYP2C9 rs1057910**							
**AA**	201	91.30	90.19–90.68	**A**	95.50	95.10–95.16	^[38][39]^
**AC**	18	8.20	8.96–9.81	**C**	4.50	4.84–4.90	
**CC**	1	0.50	0–0.36				
**CYP4F2 rs2108622**							
**AA**	8	3.60	5.68–8.89	**A**	21.10	20.50–28.31	^[26][27]^
**AG**	77	35.00	29.55–38.84	**G**	78.90	71.69–79.50	
**GG**	135	61.40	52.27–64.77				
**GGCX rs699664**							
**TT**	29	13.20	9.48	**T**	35.00	31.86	^[29]^
**TC**	96	43.60	44.74	**C**	65.00	68.14	
**CC**	95	43.20	45.77				

**Table 3:** Basic information of the cited references.

**Gene**	**SNP**	**References**	**Population**	**N(males/females)**	**Age**	**Genotypic**	**Allelic**
**VKORC1**	**rs9923231**	Miao L, et al. (2007)^[36]^	Han	178 (74/104)	Males: 57 (17–88)	**AA** 149 (83.70%)	**A** 326 (91.57%)
					Females: 53 (15–82)	**AG** 28 (15.70%)	**G** 30 (8.43%)
						**GG**1 (0.60%)	
		Wang TL, et al. (2008) ^[37]^	Han	318 (147/171)	Males:50.84±14.55 (21–78)	**AA** 274 (86.16%))	**A** 589 (92.61%)
					Females: 53.32±14.41 (19–82)	**AG** 41 (12.90%)	**G** 47 (7.39%)
						**GG** 3 (0.94%)	
	**rs7294**	Zhang Yatong, et al. (2010) ^[20]^	Han	207 (106/101)	62.8±14.4 (12–90)	**TT** 3 (1.45%)	**T** 22 (5.31%)
						**TC** 16 (7.73%)	**C** 392 (94.69%)
						**CC** 188 (90.82%)	
		Tang Xuefeng, et al. (2009) ^[21]^	Han	205 (108/97)	60.1±13.8 (-)	**TT** 2 (1.00%)	**T** 30 (7.30%)
						**TC** 26 (12.70%)	**C** 380 (92.70%)
						**CC** 177 (86.30%)	
**CYP2C9**	**rs1057910**	Huang Shengwen, et al. (2011) ^[38]^	Han	279 (-/-)	49.4±15.0 (18–87)	**AA** 253 (90.68%)	**A** 531 (95.16%)
						**AC** 25 (8.96%)	**C** 27 (4.84%)
						**CC**1 (0.36%)	
		Yu BN, et al. (2004) ^[39]^	Han	265 (138/127)	50±11 (48–62)	**AA** 239 (90.19%)	**A** 504 (95.10%)
						**AC** 26 (9.81%)	**C** 26 (4.90%)
						**CC -**	
**CYP4F2**	**rs2108622**	Li JH，et al. (2012) ^[26]^	Han	352 (123/229)	20–59	**AA** 20 (5.68%)	**A** 144 (20.50%)
						**AG** 104 (29.55%)	**G** 560 (79.50%)
						**GG**228 (64.77%)	
		Chen J, et al. (2014)^[27]^	Han	551 (308/243)	51 (43–60)	**AA** 49 (8.89%)	**A** 312 (28.31%)
						**AG** 214 (38.84%)	**G** 790 (71.69%)
						**GG**288 (52.27%)	
**GGCX**	**rs699664**	Liu Y, et al. (2011) ^[29]^	Han	970 (408/562)	47.19±11.94 (18–78)	**TT** 92 (9.48%)	**T** 618 (31.86%)
						**TC** 434 (44.75%)	**C** 1322 (68.14%)
						**CC** 444 (45.77%)	

**Figure 1:**
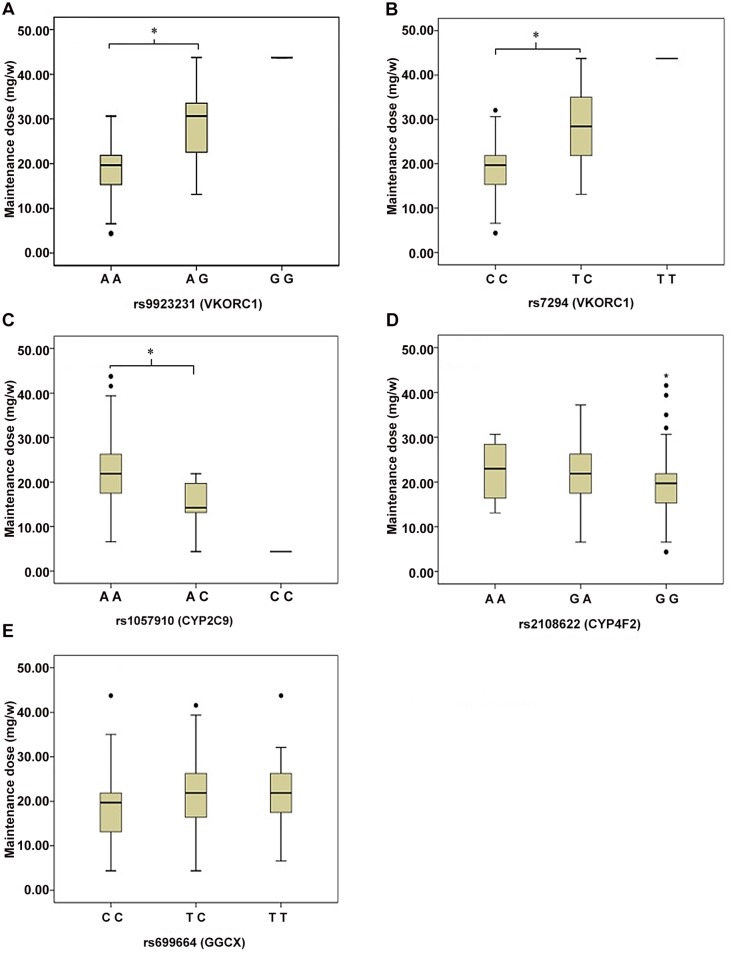
Relation between the maintenance dose and the tested genes. Panel A–E: rs9923231 (Panel A), rs7294 (Panel B), rs1057910 (Panel C), rs2108622 (Panel D), rs699664 (Panel E). Each box indicates 25 to 75 percentile of values, and the horizontal lines represent the median value of maintenance dose. *: p < 0.05 at ANOVA test.

In the 220 recruited patients, 158 individuals had the target INR (1.5–2.5). Baseline characteristics of these patients were analyzed between different genetic groups (Table 4). The age, body weight and gender ratio as the factors which might influence warfarin maintenance dose were all matched between groups. The comparison of weekly warfarin maintenance dose among those patients bearing different genotypes were shown in Fig. 1. Patients with mutational homozygote AA of *VKORC1* rs9923231 and wild homozygote CC of *VKORC1* rs7294 required a significantly lower maintenance dose than those with heterozygotes AG and TC, respectively (rs9923231: 19.21±5.66 mg/w *vs* 28.62±8.02 mg/w, *p* < 0.001; rs7294: 19.40±5.75 mg/w *vs* 27.87±8.80 mg/w, *p* < 0.001). The mean maintenance dose per week was also significantly lower in *CYP2C9* rs1057910 mutated heterozygote than in patients with the wild homozygote (15.31±5.26 mg/w *vs* 21.21±6.98 mg/w, *p* = 0.002). However, the dosage requirement difference was not observed in patients with variant genotypes of *CYP4F2* rs2108622 and *GGCX* rs699664 (*p* = 0.135 and 0.216).

**Table 4:** Baseline characteristics of patients with target INR (1.5–2.5).

**Gene**	**SNP**		**N**	**Age (year)**	***p***	**Body weight (kg)**	***p***	**Gender (Males/Females)**	***p***
**VKORC1**	**rs9923231**	**AA**	134	48.54±10.42	0.330^△^	58.75±8.67	0.793^△^	38/96	0.936^☆^
		**AG+ GG**	24	46.25±11.28		58.23±8.05		7/17	
	**rs7294**	**TT+ TC**	23	47.70±10.12	0.809^△^	59.31±8.03	0.710^△^	8/15	0.469^☆^
		**CC**	135	48.27±10.66		58.55±8.67		37/98	
**CYP2C9**	**rs1057910**	**AA**	147	48.01±10.68	0.427^△^	58.84±8.81	0.101^△^	43/104	0.730*
		**AC+ CC**	11	50.64±8.65		56.45±3.62		2/9	
**CYP4F2**	**rs2108622**	**AA+ AG**	58	48.21±9.45	0.988^△^	57.93±7.61	0.433^△^	15/43	0.579^☆^
		**GG**	100	48.18±11.19		59.12±9.09		30/70	
**GGCX**	**rs699664**	**TT**	24	46.63±10.03	0.521^◆^	55.52±8.58	0.109^◆^	7/17	0.936^☆^
		**TC+ CC**	134	48.47±10.65		59.27±8.448		38/96	

Data of age and body weight represented as mean ± SD, and the continuous variables for rs9923231, rs7294, rs1057910, rs2108622 are normalized, except rs699664.

^△^: normalized data were analyzed by Independent-Samples T test;

^◆^: non-normalized data were analyzed by Mann-Whitney U test;

^☆^: data analyzed by χ^2^-test;

*: data analyzed by fisher exact test.

**Figure 2:**
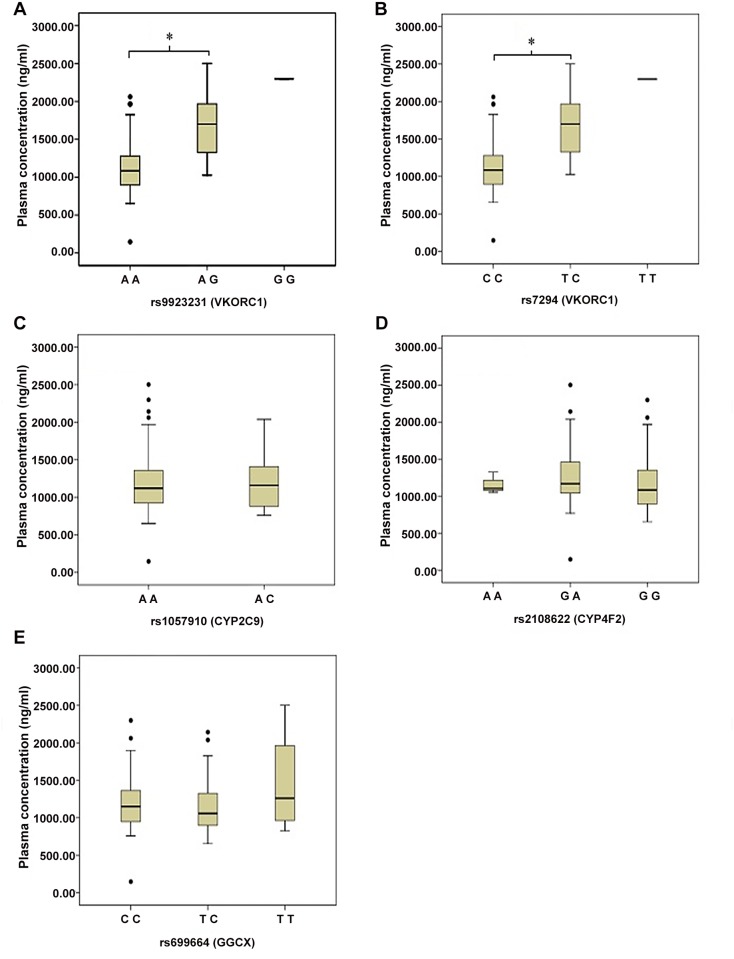
Relation between the plasma concentration and the tested genes. Panel A—E: rs9923231 (Panel A), rs7294 (Panel B), rs1057910 (Panel C), rs2108622 (Panel D), rs699664 (Panel E). Each box indicates 25 to 75 percentile of values, and the horizontal lines represent the median value of plasma concentration. *: p < 0.05 at ANOVA test.

Only 130 plasma samples were obtained successfully at 12 hours after the administration of the last dose of warfarin. The plasma warfarin concentrations of these samples were detected. Fig. 2 showed the comparison of plasma concentration among those patients with INR (1.5–2.5) (n = 92) bearing different genotypes. The polymorphisms of *VKORC1* rs9923231 and rs7294 were also associated with a significant reduction of warfarin plasma concentration (rs9923231: 1117.29±323.23 ng/ml *vs* 1675.73±431.09 ng/ml, *p* < 0.001; rs7294: 1117.29±323.23 ng/ml *vs* 1675.73±431.09 ng/ml, *p* < 0.001).

In addition, the effects of target SNPs on the warfarin maintenance dose variability and plasma concentration were analyzed (Table 5). Age, body weight, gender, rs9923231, rs1057910, rs7294, rs2108622 and rs699664 were included as the independent variables. For maintenance dose, rs9923231 and rs1057910 had significant effects on it (rs9923231: coefficient was 1.398, p < 0.001; rs1057910: coefficient was-0.994, p < 0.001). Both them could explain about 32.0% of the variability in warfarin maintenance dose. For plasma concentration, rs9923231 was eliminated because of the colinearity. Only rs7294 had significant effects on plasma concentration of warfarin (coefficient was 0.527, p < 0.001) and it could explain 26.7% of the variability in plasma concentration.

**Table 5:** The effects of rs9923231, rs7294, rs1057910, rs2108622, rs699664 on warfarin maintenance dose and plasma concentration.

**SNP**	**Genotype**	**N**	**Maintenance dose(mg/w)**	Coefficient*	p*	Adjusted R^2^*	**N**	**Plasma concentration (ng/ml)**	**Coefficient^△^**	***p^△^***	**Adjusted R^2△^**
		158				0.320	92				0.267
**VKORC1 rs9923231**	**AA**	134	19.21±5.66				79	1117.29±323.23			
	**AG**	23	28.62±8.02	1.398	<0.001		12	1675.73±431.09	-	-	
	**GG**	1	43.75				1	2298.50			
**VKORC1 rs7294**	**TT**	1	43.75				1	2298.50			
	**TC**	22	27.87±8.80	-	0.297		12	1675.73±431.09	0.527	<0.001	
	**CC**	135	19.40±5.75				79	1117.29±323.23			
**CYP2C9 rs1057910**	**AA**	147	21.21±6.98				86	1200.78±399.18			
	**AC**	10	15.31±5.26	-0.994	<0.001		6	1234.23±470.14	-	0.326	
	**CC**	1	4.38				-	-			
**CYP4F2 rs2108622**	**AA**	4	22.42±7.66				3	1161.07±144.99			
	**AG**	54	22.21±6.40	-	0.056		27	1284.63±475.94	-	0.203	
	**GG**	100	19.87±7.39				62	1169.43±372.61			
**GGCX rs699664**	**TT**	24	22.53±7.34				10	1405.36±560.57			
	**TC**	72	21.06±7.09	-	0.134		50	1158.14±352.51	-	0.143	
	**CC**	62	19.66±7.01				32	1209.76±410.63			

Analyzed by stepwise multiple linear regression.

*:the dependent variable was maintenance dose and the independent variables included age, body weight, gender, rs9923231, rs1057910, rs7294, rs2108622 and rs699664;

p *:showed the effects of 5 SNPs on the maintenance dose;

Adjusted R2*:showed the contribution of rs9923231and rs1057910 to the variability in warfarin maintenance dose.

^△^:the dependent variable was plasma concentration and the independent variables included age, body weight, gender, rs9923231, rs1057910, rs7294, rs2108622 and rs699664.

p ^△^:showed the effects of 5 SNPs on the plasma concentration;

Adjusted R2^△^: showed the contribution of rs7294 to the plasma concentration.

The relationship between plasma concentration and maintenance dose was evaluated among patients carrying polymorphism variants to explore the effect of rs1057910 (CYP2C9) on the pharmacokinetics of warfarin (Table 6). For the low-dosage group (<17.5 mg/w) and the middle-dosage group (17.5–26.25 mg/w), slightly higher plasma concentration were required by variant heterozygotes AC than wild homozygotes AA though the statistical difference were not significant in this study. In addition, the remaining four polymorphism loci: rs9923231, rs7294 in *VKORC1*, rs2108622 in *CYP4F2* and rs699664 in *GGCX* associated with the pharmacodynamics of warfarin were assessed (Table 7). Patients were grouped according to INR and genotypes. For patients with allele G of rs9923231 and allele T of rs7294, higher plasma concentration was needed to achieve the similar goal INR. No significant effect for rs2108622 and rs699664 was observed.

**Table 6:** The relationship between plasma concentration and maintenance dose in patients carrying polymorphism variants of rs1057910.

		**Maintenance dose(mg/w)**		
**Plasma concentration(ng/ml)**	**N**	**<17.5 (n = 37)**	**[17.5–26.25) (n = 64)**	**≥26.25 (n = 29)**
**rs1057910 (CYP2C9)**	130			
**AA**	119	1022.59±290.60	1115.45±334.71	1399.10±538.73
**AC**	11	1040.62±426.11	1288.86±464.00	-
**CC**	-	-	-	-
***p***		0.967	0.284	-

**Table 7:** The relationship between plasma concentration and INR in patients carrying polymorphism variants of rs9923231, rs7294, rs2108622 and rs699664.

		**INR**		
**Plasma concentration(ng/ml)**	**N**	**<1.5 (n = 27)**	**[1.5–2.5] (n = 92)**	**>2.5 (n = 11)**
**rs9923231 (VKORC1)**	130			
**AA**	109	913.66±266.13	1117.29±323.23	1009.54±339.53
**AG**	20	1528.43±623.17	1675.73±431.09	1328.80±15.84
**GG**	1	-	2298.50	-
***p***		0.017	<0.001	0.234
**rs7294 (VKORC1)**				
**TT**	1	-	2298.50	-
**TC**	20	1528.43±623.17	1675.73±431.09	1328.80±15.84
**CC**	109	913.66±266.13	1117.29±323.23	1009.54±339.53
***p***		0.017	<0.001	0.234
**rs2108622 (CYP4F2)**				
**AA**	6	1557.13±793.26	1161.07±144.99	-
**AG**	39	950.26±354.96	1284.63±475.94	1293.07±233.87
**GG**	85	1008.92±375.03	1169.43±372.61	983.04±331.70
***p***		0.102	0.458	0.177
**rs699664 (GGCX)**				
**TT**	36	848.00	1209.76±410.63	992.43±416.99
**TC**	67	1045.92±401.85	1158.14±352.51	1186.10±245.59
**CC**	27	1068.46±503.82	1405.36±560.57	945.23±429.05
***p***		0.898	0.206	0.596

## Discussion

In this study, the effect of genetic factors on the pharmacokinetics and pharmacodynamics of warfarin in Chinese population was investigated. *VKORC1* and *CYP2C9* are identified as major determinants to warfarin dose variability, and about 30% to 40% of dose variation could be attributed to those two genes [8–10]. In the *VKORC1* gene, rs9923231 (-1639 G>A) is the most common SNP. Allelic frequency was found to be the highest in the GG genotype and lowest in the AA genotype with the Caucasian population [3]. However, in the Chinese population, AA became the majority genotype [17]. Our study confirmed this finding. rs9934438 (1173 C>T) and rs7294 (3730 G>A) were two other familiar SNPs in *VKORC1*. rs9934438 was in strong linkage disequilibrium with rs9923231, which had been reported in several ethnic groups [18, 19]. rs7294 was rarely reported in Chinese population, and the relation between it and rs9923231 remained unclear. Our population had the similar allele and genotype frequencies of rs7294 compared with other Chinese populations [20, 21]. Furthermore, strong linkage disequilibrium was also found between these two SNPs in our study. rs1057910 (CYP2C9*3) and rs1799853 (CYP2C9*2) were the most common polymorphism loci in *CYP2C9* gene. However, a major difference was observed between the Caucasians and Chinese in the allelic frequency of rs1799853. About 10–15% of Caucasians carried this mutant allele, but is absent in most Chinese [3]. This study only genotyped for rs1057910, and there was no difference with other Chinese subjects.

CYP4F2 is the vitamin K oxidase involved in the metabolism of vitamin K, and the variations of *CYP4F2* may reduce the capacity to metabolize vitamin K [23]. About 30% of Caucasian population and 17% of Asians carried the minor allele of rs2108622, which could explain 2–5% of the variance in dose [12, 23–25]. The studies aimed at Chinese Han population had reported that 20.50%-28.31% of the population had the minor allele A of rs2108622 [26, 27]. We found the similar proportions in our study (21.10%).

GGCX catalyzes the post-translational modification of vitamin K-dependent proteins, and is one of the candidate genes in individual differences of response to warfarin [28]. The polymorphism of rs699664 was associated with 4%–7% of the dosage difference. Liu Y et al. reported that CC was the primary genotype in Chinese population (45.77%) though it was slightly higher than that of TC (44.74%) [29]. We found that the frequencies of TC and CC were more similar (43.60% and 43.20%)

As the target gene for warfarin, the mutational allele A of rs9923231 in VKORC1 could increase warfarin sensitivity, which induced a lower dose requirement [10]. Conversely, higher dosage was required by the mutational allele T of rs7294 patients with respect to subjects carrying the wild-type allele [30]. The polymorphisms of rs1057910 are associated with reduced metabolism of warfarin. Thus, individuals carrying the allele C have lower maintenance dose requirements than those carrying the wild-type allele [31]. Those heterozygous for the mutation allele require 34%–38% lower daily maintenance doses than homozygous wild-type individuals [32–34]. We confirmed this dosage difference brought by these three SNPs, and further confirmed that 32.0% of the dosage variability came from rs9923231 and rs1057910. Furthermore, the effect of *CYP4F2* rs2108622 remained controversial. Mc Donald MG et al. maintained that patients with rs2108622 polymorphism were likely to have elevated hepatic levels of vitamin K, and a higher warfarin dose might be required to obtain the same anticoagulant response [22]. Liang et al. also found that CYP4F2 could explain 3.9% of the dose variation [13]. However, another study by Lee et al. found that this polymorphism didn’t significantly influence warfarin dosage in Chinese [35]. We obtained the similar results to Lee et al. Few studies refered to rs699664 in *GGCX*. In the study carried by Chen J et al, they could not confirm the previous associations of rs699664 with warfarin dose in Chinese population [27]. Likewise, we also did not find significant association between this SNP and daily maintenance dose. For plasma concentration, few studies had mentioned the influence from SNPs. In our study, the effects from 5 SNPs were not remarkable. Although the allele A of rs9923231 and C of rs7294 also brought lower plasma concentration, only rs7294 had significant influence on the plasma concentration and its contribution was 26.7%.

The relationship between plasma concentration and INR was evaluated to investigate the effect of rs9923231, rs7294, rs2108622 and rs699664 on the pharmacodynamics of warfarin. Significant association was found in patients with polymorphisms of rs9923231 and rs7294 in *VKORC1* in this study. For a target INR, patients with allele A of rs9923231 and allele C of rs7294 required lower plasma concentration. Notwithstanding, for rs2108622 and rs699664, no significant trend was observed, a larger sample size may be needed. Analogously, rs1057910 in *CYP2C9* participated in the metabolism of warfarin. Its pharmacokinetics effect was assessed by maintenance dose and plasma concentration. Patients with allele A had a lower plasma concentration under similar maintenance dose of warfarin, although this difference was not statistically significant. Further studies are needed to confirm this finding.

In conclusion, there is no doubt about the ethnic characteristic of genetic polymorphisms. For warfarin therapy, the fixed-dose for all patients might not be appropriate. The important contribution of genetic polymorphism to warfarin dosage variability has already attracted attention. By exploring the relevant genetic variants, this study contributes to a broader and richer insight into the pharmacodynamics and pharmacokinetics of warfarin in Chinese population. A better understanding of the genetic variants in individuals can be the foundation for warfarin dosing algorithm and would facilitate a more reasonable and effective use.
